# Development of sandwich ELISA and lateral flow strip assays for diagnosing clinically significant snakebite in Taiwan

**DOI:** 10.1371/journal.pntd.0007014

**Published:** 2018-12-03

**Authors:** Chien-Chun Liu, Jau-Song Yu, Po-Jung Wang, Yung-Chin Hsiao, Chien-Hsin Liu, Yen-Chia Chen, Pei-Fang Lai, Chih-Po Hsu, Wen-Chih Fann, Chih-Chuan Lin

**Affiliations:** 1 Molecular Medicine Research Center, Chang Gung University, Tao-Yuan, Taiwan; 2 Department of Cell and Molecular Biology, College of Medicine, Chang Gung University, Tao-Yuan, Taiwan; 3 Liver Research Center, Chang Gung Memorial Hospital, Linkou, Tao-Yuan, Taiwan; 4 Research Center for Food and Cosmetic Safety, Research Center for Chinese Herbal Medicine, College of Human Ecology, Chang Gung University of Science and Technology, Tao-Yuan, Taiwan; 5 Department of Emergency Medicine, Chang Gung Memorial Hospital, Tao-Yuan, Taiwan; 6 Center for Research, Diagnostics and Vaccine Development, Centers for Disease Control, Ministry of Health and Welfare, Taipei, Taiwan; 7 Department of Emergency medicine, Taipei Veterans General Hospital, Taipei, Taiwan; 8 Department of Emergency Medicine, School of Medicine, National Yang-Ming University, Taipei, Taiwan; 9 Department of Emergency, Buddihist Tzu Chi Hospital, Hualien, Taiwan; 10 Department of Trauma and Emergency Surgery, Chang Gung Memorial Hospital, Chang Gung University, Tao-Yuan, Taiwan; 11 Department of Emergency Medicine, Chia-Yi Chang Gung Memorial Hospital, Chiayi, Taiwan; Texas A&M University Kingsville, UNITED STATES

## Abstract

Taiwan is an island located in the south Pacific, a subtropical region that is home to 61 species of snakes. Of these snakes, four species—*Trimeresurus stejnegeri*, *Protobothrops mucrosquamatus*, *Bungarus multicinctus* and *Naja atra*—account for more than 90% of clinical envenomation cases. Currently, there are two types of bivalent antivenom: hemorrhagic antivenom against the venom of *T*. *stejnegeri* and *P*. *mucrosquamatus*, and neurotoxic antivenom for treatment of envenomation by *B*. *multicinctus* and *N*. *atra*. However, no suitable detection kits are available to precisely guide physicians in the use of antivenoms. Here, we sought to develop diagnostic assays for improving the clinical management of snakebite in Taiwan. A two-step affinity purification procedure was used to generate neurotoxic species-specific antibodies (NSS-Abs) and hemorrhagic species-specific antibodies (HSS-Abs) from antivenoms. These two SSAbs were then used to develop a sandwich ELISA (enzyme-linked immunosorbent assay) and a lateral flow assay comprising two test lines. The resulting ELISAs and lateral flow strip assays could successfully discriminate between neurotoxic and hemorrhagic venoms. The limits of quantification (LOQ) of the ELISA for neurotoxic venoms and hemorrhagic venoms were determined to be 0.39 and 0.78 ng/ml, respectively, and the lateral flow strips were capable of detecting neurotoxic and hemorrhagic venoms at concentrations lower than 5 and 50 ng/ml, respectively, in 10–15 min. Tests of lateral flow strips in 21 clinical snakebite cases showed 100% specificity and 100% sensitivity for neurotoxic envenomation, whereas the sensitivity for detecting hemorrhagic envenomation samples was 36.4%. We herein presented a feasible strategy for developing a sensitive sandwich ELISA and lateral flow strip assay for detecting and differentiating venom proteins from hemorrhagic and neurotoxic snakes. A useful snakebite diagnostic guideline according to the lateral flow strip results and clinical symptoms was proposed to help physicians to use antivenoms appropriately. The two-test-line lateral flow strip assay could potentially be applied in an emergency room setting to help physicians diagnose and manage snakebite victims.

## Author summary

Snakebite is a public health issue that causes life-threatening medical emergencies. Rapid diagnosis of snakebite in the clinic is a critical necessity in many tropical and subtropical countries, where various venomous snakes are common. Venoms from different snake species contain distinct protein components that require treatment with different antivenoms. However, given the similarity in clinical symptoms among some snake envenomations, it is often challenging for physicians to precisely define the snake species responsible for envenomation. Thus, a reliable method or assay for rapidly diagnosing envenoming species is urgently needed. Here, we present a two-step affinity purification procedure for generating species-specific antibodies (SSAbs) from antivenom, followed by the development of a sandwich ELISA (enzyme-linked immunosorbent assay) and lateral flow strip assay using these SSAbs. This feasible and cost-effective strategy allowed us to develop workable assays for distinguishing between venom proteins from hemorrhagic and neurotoxic snakes in Taiwan. The usefulness of this strategy was demonstrated in the clinic, where both diagnostic assays were shown capable of detecting venoms in blood samples from snakebite patients. Together with the observation of clinical symptoms, the two-test-line lateral flow strip assay is potentially applicable in an emergency room setting to improve snakebite diagnosis and management.

## Introduction

Envenoming resulting from snakebites is a significant public health issue in many regions of the world, particularly in tropical and subtropical countries and some poor rural communities [1]. An estimated 1,800,000–2,700,000 envenoming cases and 81,410–137,880 associated deaths occur each year globally owing to snakebite [2]. The regions with the highest burden are South Asia, Southeast Asia, and Africa [2, 3].

Administration of antivenom is the standard treatment for snake envenomation. In most countries, multiple types of antivenom are clinically available, but uncertainty regarding the appropriate antivenom to use in any given situation remains an important issue. To date, the species responsible for envenomation of snakebite victims referred for medical treatment is initially identified primarily based on the shape of the wound or identification of dead snakes brought to the hospital. Thereafter, the physician monitors local symptoms to confirm which antivenom should be used. However, some clinical symptoms caused by envenomation are similar among species, and non-venomous snakes are often responsible for the patient’s snakebite [4]. Additionally, physicians are often misled by incorrect descriptions of the snake by victims or their family members [5]. Identification of venomous snake species is important for optimal clinical management, because it allows physicians to use the correct antivenom for effective treatment, thereby improving patients’ prognosis and preventing the waste of expensive antivenoms and exposing victims to antivenom-induced adverse reactions [6]. Although identification of snake species is important for the management of snakebite-related injuries worldwide, there are currently no developed standard platforms or guidelines for snakebite diagnosis globally.

Detection of venom proteins using antibodies is a simple and effective approach for identifying the species responsible for snakebite. To date, various immunoassays for detecting venom proteins in body fluids have been described [7–13], including radioimmunoassay [14], agglutination assays [9, 15], enzyme-linked immunosorbent assays (ELISAs) [10–12, 16, 17], and fluorescence immunoassays [18, 19]. In addition to immunoassays, immunology-based biosensors have been explored for detection of snakebite [20, 21]. ELISAs and lateral flow assays [22, 23] are arguably the best choice of immunoassays for snakebite identification. ELISAs, the most common and general immunoassays in clinical use, are sensitive to their target at pictogram per milliliter levels [18]. Although the antibodies are relatively costly, ELISA devices and reagents are affordable for routine diagnosis. Compared with ELISAs, lateral flow assays offer advantages in terms of detection time and required equipment: it takes only ~5–20 min to obtain assay results and no supporting instrumentation is needed [24, 25]. Although lateral flow assays mainly provide qualitative results, their simple design and operation compared with quantitative ELISAs make them the most user-friendly for the public, allowing rapid adoption in rural countries.

Snake venoms contain many proteins, and closely related snake species have some of the same or similar venom components, causing cross-reactions in immunoassays applied to detect venom proteins [11, 12, 26]. The venom antigens responsible for the observed cross-reactivity would further cause ambiguities and false-positive results in snake species detection [11, 27]. Hence, the direct use of polyclonal antibodies against whole venoms for snake species detection is inappropriate, and elimination of cross-reactive antibodies is critical for generating an immunoassay with high specificity for discriminating snake species [11, 12, 28]. Solving the problem of cross-reaction and improving the specificity of immunoassays might most efficiently be achieved through purification of species-specific antibodies (SSAbs) on affinity columns immobilized with venom proteins cross-reactive to the polyclonal antibodies or antisera [11, 12].

Six venomous snakes—*Deinagkistrodon acutus*, *Trimeresurus stejnegeri*, *Protobothrops mucrosquamatus*, *Daboia russelii formosensis*, *Bungarus multicinctus* and *Naja atra*—are indigenous to Taiwan, a subtropical island in East Asia [29]. Four kinds of antivenom had been produced by the Vaccine Center, Center for Disease Control, Taiwan, to treat envenomation by these six venomous snakes and effectively limit snakebite mortality [30]. Freeze-dried hemorrhagic antivenom (FHAV) is used to treat envenomation by *T*. *stejnegeri* and *P*. *mucrosquamatus*, and freeze-dried neurotoxic antivenom (FNAV) neutralizes venom of *B*. *multicinctus* and *N*. *atra*. Envenomation by the other two snake species is treated by monovalent antivenoms. A population-based study of venomous snakebites in Taiwan from 2005 to 2009 reported a total of 4647 snakebite cases, of which 380 (8.1%) received at least two types of antivenoms, mainly because of similarities in the clinical presentations of different snakebites and the inability of some patients to identify the culprit snake [31]. In some studies, such unidentified cases accounted for 12–45% of total cases [32–35]. In addition, according to a clinical survey of antivenom usage in Taiwan, more than 99% of snakebite patients that had received FHAV or FNAV treatment were rescued [36], indicating that most snakebite cases in Taiwan represent envenomation by *T*. *stejnegeri*, *P*. *mucrosquamatus*, *B*. *multicinctus* or *N*. *atra*. Unfortunately, there have been very few efforts to develop sensitive assays for detecting snake venom in Taiwan. Currently, only one ELISA-based blood assay has been developed to detect the *N*. *atra* venom, but it is not commercially available [7], and no laboratory test can be used to identify other types of venoms.

In the present study, we designed a workflow to develop immunoassays for snakebite detection based on clinical antivenom usage in Taiwan. We used FHAV and FNAV as resources for purification of hemorrhagic species-specific antibodies (HSS-Ab) and neurotoxic species-specific antibodies (NSS-Ab), and applied these two critical reagents to develop ELISAs and lateral flow strip assays. These assays hold the potential for use in identification of snake species responsible for snakebites in Taiwan.

## Materials and methods

### Snake venoms and hyper-immunized horse plasma

Lyophilized venoms of *T*. *stejnegeri*, *P*. *mucrosquamatus*, *B*. *multicinctus* and *N*. *atra* were obtained from the Center for Disease Control, R.O.C (Taiwan). The venoms were collected from several adult specimens, then freeze-dried and stored at -20°C before use. Hemorrhagic venom (*T*. *stejnegeri* and *P*. *mucrosquamatus*)-immunized and neurotoxic venom (*B*. *multicinctus* and *N*. *atra*)-immunized horse plasma were also donated by the Center for Disease Control, R.O.C (Taiwan). The plasma was stored at -80°C before use.

### Affinity purification of SSAbs

For coupling of venom proteins onto Sepharose beads, CNBr-activated Sepharose 4B was swollen in 1.0 mM HCl (pH 3.0), then incubated with 10 mg hemorrhagic or neurotoxic snake venoms dissolved in coupling buffer (0.1 M NaHCO_3_ pH 8.3) overnight at 4°C on a round rotator. After washing with coupling buffer, any remaining active sites on beads were blocked by incubating overnight at 4°C with blocking buffer (1.0 M diethanolamine pH 8.0) on a rotator. The beads were then alternately washed three times with an acidic buffer (0.1 M C_2_H_3_NaO_2_ pH 4.0, 0.5 M NaCl) and basic buffer (0.1 M Tris pH 8.0, 0.5 M NaCl) and packed into a column, The resulting venom affinity columns were equilibrated with binding buffer (10 mM Tris-HCl pH 7.5) and stored at 4°C before use.

To purify HSS-Ab, 2 ml FHAV was diluted in 30 ml of binding buffer and the diluted sample was pumped into the neurotoxic venom affinity column at 4°C for 3 h. The flow-through fraction was then pumped into the hemorrhagic venom affinity column at 4°C for another 3 h. The hemorrhagic venom affinity column was then washed with 60 ml binding buffer and 60 ml wash buffer (10 mM Tris-HCl pH 7.5,0.5 M NaCl). After washing, each affinity column was eluted with 20 ml of acidic (100 mM glycine pH 2.5) or basic (100 mM triethylamine pH 11.5) elution buffer, and eluted fractions (1 ml/fraction) were collected into microcentrifuge tubes containing 100 μl of neutralized buffer (1.5 M Tris-HCl pH 8.0). Finally, all eluted fractions were pooled, concentrated, and exchanged into phosphate-buffered saline (PBS) by dialysis overnight. The concentrated antibodies in PBS were diluted with an equal volume of glycerol and stored at -20°C. Similar protocol was used to purify NSS-Ab from 2 ml FNAV, in which the diluted FNAV was passed through the hemorrhagic venom affinity column first, and the flow-through fraction containing NSS-Ab was further purified using the neurotoxic venom affinity column.

### Indirect ELISA assays

Snake venom proteins (100 ng) were diluted in 100 μl PBS and coated onto 96-well polystyrene microplates (Corning, USA) by incubating at 4°C overnight. The plates were washed six times with 200 μl of PBST (PBS contain 0.1% Tween-20) and blocked by incubating with 200 μl of 1% ovalbumin in PBS at room temperature for 2 h. After washing wells six times with PBST, antivenom or purified Ab (1 mg/ml) was serial diluted (from 1:2000 to 1:16000) and added to individual wells, then the plate was incubated at room temperature for 2 h. Wells were again washed six times with PBST, and then alkaline phosphatase-conjugated anti-horse IgG antibody (Santa Cruz Biotechnology, USA) was added to each well and the plate was incubated at room temperature for 1 h. After washing six times with PBST, the substrate 4-methyl umbelliferyl phosphate (100 μM, 100 μl/well; Molecular Probes) was added to each well, and fluorescence was measured with a SpectraMax M2 microplate reader (Molecular Devices, USA) at excitation and emission wavelengths of 355 and 460 nm, respectively.

### Western blot analysis

Snake venom proteins (5 μg) were separated by sodium dodecyl sulfate-polyacrylamide gel electrophoresis (SDS-PAGE), transferred onto PVDF (polyvinylidene difluoride) membranes (Millipore, USA), and probed with antivenom or purified Ab. Immunoreactive proteins in PVDF membranes were detected by incubating for 1 h with the appropriate alkaline phosphatase-conjugated anti-horse IgG antibodies (Santa Cruz Biotechnology, USA) and visualized using the CDP-Star Western Blot Chemiluminescence Reagent (PerkinElmer, USA).

### Biotinylation of SSAbs

Antibodies were biotinylated using a Lightning-Link biotinylation kit (Innova Biosciences, USA) according to the protocol provided by the manufacturer. Briefly, 100 μl of SSAb (2 mg/ml) was mixed with 10 μl of modifier reagent, then added to the tube containing biotinylation powder and incubated for 15 min in the dark. After the biotinylation reaction, 10 μl of quencher reagent was added and the reaction mixture was stored at -20°C until use.

### Sandwich ELISA assays

SSAb (100 μl at 2 mg/ml), diluted 1:1000 in PBS, was coated onto 96-well polystyrene microplates. Thereafter, wells were blocked by incubating with 1% bovine serum albumin (BSA) in PBS for 1 h, then washed six times with 200 μl PBST. Test samples (100 μl) were added into individual wells and incubated at room temperature for 2 h. After washing six times with PBST, 100 μl of biotin-labeled SSAb, diluted 1:16000 in PBS, was added and plates were incubated for 2 h. Plates were again washed six times with PBST, then alkaline phosphatase-conjugated streptavidin was added and allowed to interact with biotin. The alkaline phosphatase substrate, 4-methyl umbelliferyl phosphate (100 μM, 100 μl/well), was then added to each well, and fluorescence was measured with a SpectraMax M5 microplate reader at excitation and emission wavelengths of 355 and 460 nm, respectively.

### Snakebite animal model and plasma sample collection

Experiments were performed on male 7-wk-old littermate mice (C57BL/6Narl strain). Mice were maintained under specific pathogen-free conditions with a 12:12 h light-dark cycle at a temperature of 22°C and a humidity level of 60–70%. Animals had ad libitum access to food and water. Mice (n = 3/group) within a defined weight range (20–25 g) were subcutaneously (*B*. *multicinctus* and *N*. *atra* venom) or intraperitoneally (*T*. *stejnegeri* and *P*. *mucrosquamatus* venom) injected with a precise 0.1 ml volume of sterile saline solution containing a minimal lethal dose (MLD) of venom. Blood samples from each mouse were collected using a heparinized capillary blood collection system (Kent Scientific, USA) 0.5, 1, 1.5 and 2 h after venom injection. Collected blood was centrifuged at 3000 × g for 20 min. The resulting supernatant (plasma) was collected into a microcentrifuge tube and stored at -80°C before use.

### Preparation of colloidal gold-labeled SSAbs

A colloidal gold (40 nm) solution (REGA Biotechnology Inc., Taipei, Taiwan) was adjusted to pH 8.0 with 0.1 M potassium carbonate. The optimal concentration of SSAb (10 mg) was added to 2 ml of colloidal gold solution and incubated at room temperature for 10 min with gentle mixing. The mixture was blocked by incubating with 0.5 ml of 5% BSA in PBS at room temperature for 15 min with gentle mixing, and then centrifuged at 10,000 × g at 4°C for 30 min. The gold pellets were suspended in PBST containing 1% BSA, and washed by repeated centrifugation and suspension in the same solution. The final precipitates were suspended in 1 ml PBST containing 1% BSA and stored at 4°C until use.

### Development of lateral flow strips

The strips were manufactured by REGA Biotechnology Inc. (Taipei, Taiwan). Nitrocellulose membranes, sample pads, conjugate pads and absorbent pads were all from REGA Biotechnology Inc. Conjugate pads were saturated with HSS-Ab–or NSS-Ab–conjugated colloidal gold, then dried at 37°C for 1 h before assembling. The nitrocellulose membrane was pasted to the cardboard, after which conjugated and absorbent pads were also pasted to the cardboard such that they overlapped with each side of the nitrocellulose membrane by about 2 mm. The sample pad was also laid over the absorbent pad (2 mm overlap) and pasted onto the cardboard. The AGISMART RP-1000 rapid test immuno-strip printer (REGA Biotechnology Inc.) was used to dispense HSS-Abs and NSS-Abs (2 mg/ml) onto hemorrhagic and neurotoxic test lines, respectively, and goat anti-horse IgG antibody (2 mg/mL) (REGA Biotechnology Inc.) onto the control line on the nitrocellulose membrane. The distance between each line was 5 mm. The strips were prepared and assembled in a low-humidity environment, packaged into an aluminum pouch, and stored at room temperature before use.

### Clinical sample collection

Patients with suspected snakebite were admitted directly to the Emergency Departments of Taipei Veteran General Hospital, Linkou Chang Gung Memorial Hospital, Chiayi Chang Gung Memorial Hospital or Hualien Tzu Chi Hospital, and did not receive antivenom treatment before being enrolled in this study. After obtaining signed, informed consent forms from patients, 5 ml of blood was collected in SST blood collection tubes (BD, Franklin Lakes, New Jersey, USA) and centrifuged at 4°C for 10 min to obtain serum samples. A 100–200 μl aliquot of serum sample was immediately applied to lateral flow strip test in the emergency room, and results were determined by clinical physicians. The remainder of each sample was sent to the laboratory in Chang Gung University and stored at -80°C. All samples were re-analyzed using the lateral flow strip test in the laboratory to confirm emergency room result; samples were also analyzed by sandwich ELISA to measure the concentrations of venom proteins.

### Venom detection with lateral flow strips

Each serum sample (100–200 μl) was diluted with 1 volume of reaction buffer (100 mM borax, 250 nM polyvinylpyrrolidone (PVP)-40 and 1% Triton X-100) in a microcentrifuge tube. The strips were directly soaked in the samples, and results were recorded after a 10-min reaction.

### Agreement between lateral flow strip and sandwich-ELISA methods

The Cohen's kappa coefficient (κ) statistic [37, 38] was used to assess the strength of inter-method agreement for diagnosis results. The value of kappa coefficient statistic over 0.75, between 0.75 to 0.40, or below 0.40 indicates excellent agreement, good to fair agreement, and poor agreement, respectively [39, 40].

### Ethics statement

All clinical serum samples were collected and obtained at Taipei Veteran General Hospital, Linkou Chang Gung Memorial Hospital, Chiayi Chang Gung Memorial Hospital or Hualien Tzu Chi Hospital from February 2017 to February 2018. All study subjects are adult participants and signed an informed consent form approved by the Institutional Review Board (IRB) of Taipei Veteran General Hospital (Approval No: 2017-06-013BCF) and Linkou Chang Gung Memorial Hospital (Approval No: 201800098B0) permitting the use of plasma samples for this study. Experiments involving the care, bleeding, and injection of mice with various venoms were reviewed and approved by the Institutional Animal Care and Use Committee of Chang Gung University (Permit Number: CGU14-024). The protocol for mouse studies was based on guidelines provided by the Council for International Organizations of Medical Sciences (CIOMS) [41].

## Results

### Immunoreactivity and cross-reactivity among four venoms and two antivenoms

**Fig 1:**
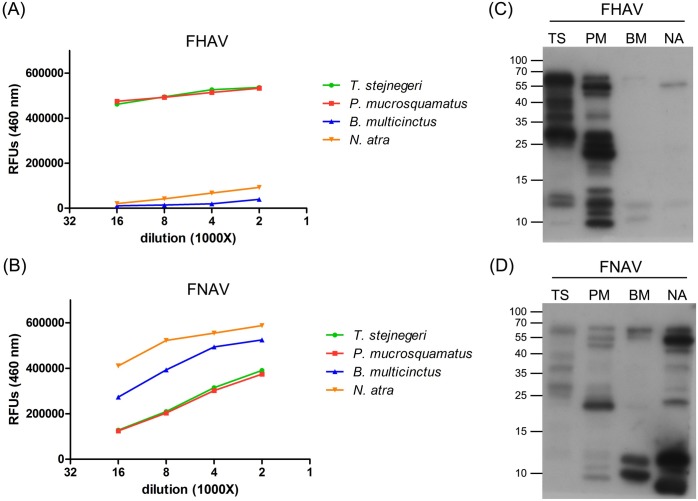
The cross-reactivity of FHAV and FNAV. (A, B) FHAV (A) and FNAV (B) were serially diluted from 1:2000 to 1:16000 and used as primary antibodies to detect four snake venoms. The assay was performed in duplicate, and results are presented as average values in relative fluorescence units (RFUs). (C, D) Venom proteins (5 μg) from *T*. *stejnegeri* (TA), *P*. *mucrosquamatus* (PM), *B*. *multicinctus* (BM), and *N*. *atra* (NA) were resolved by SDS-PAGE on 15% gels, transferred to PVDF membranes, and probed with FHAV (C) and FNAV (D) (2 μg/ml) as primary antibodies.

To assess the cross-reactivity among four venoms and two antivenoms, we performed indirect ELISAs and immunoblotting. The results of indirect ELISAs showed that cross-reactivity of FHAV towards *B*. *multicinctus* and *N*. *atra* venom was very low (**Fig 1A**); however, FNAV strongly cross-reacted with *T*. *stejnegeri* and *P*. *mucrosquamatus* venom (**Fig 1B**). Cross-reaction signals increased gradually with increases in antivenom concentration, and both antivenoms showed stronger reactivity toward homologous venoms than heterologous venoms. As shown in Western blot profiling data, FHAV primarily cross-reacted with protein bands in the high molecular weight region (55–70 kDa) of *N*. *atra* venom (**Fig 1C**), whereas FNAV cross-reacted with multiple bands in *T*. *stejnegeri* and *P*. *mucrosquamatus* venoms, predominantly towards protein bands in the 15–25 kDa range in *P*. *mucrosquamatus* venom (**Fig 1D**). A comparison of the protein profiles of the four venoms (**S1 Fig**) showed that, generally, most venom components of these venoms were recognized by the corresponding homologous antivenom.

### HSS-Ab and NSS-Ab specificity

**Fig 2:**
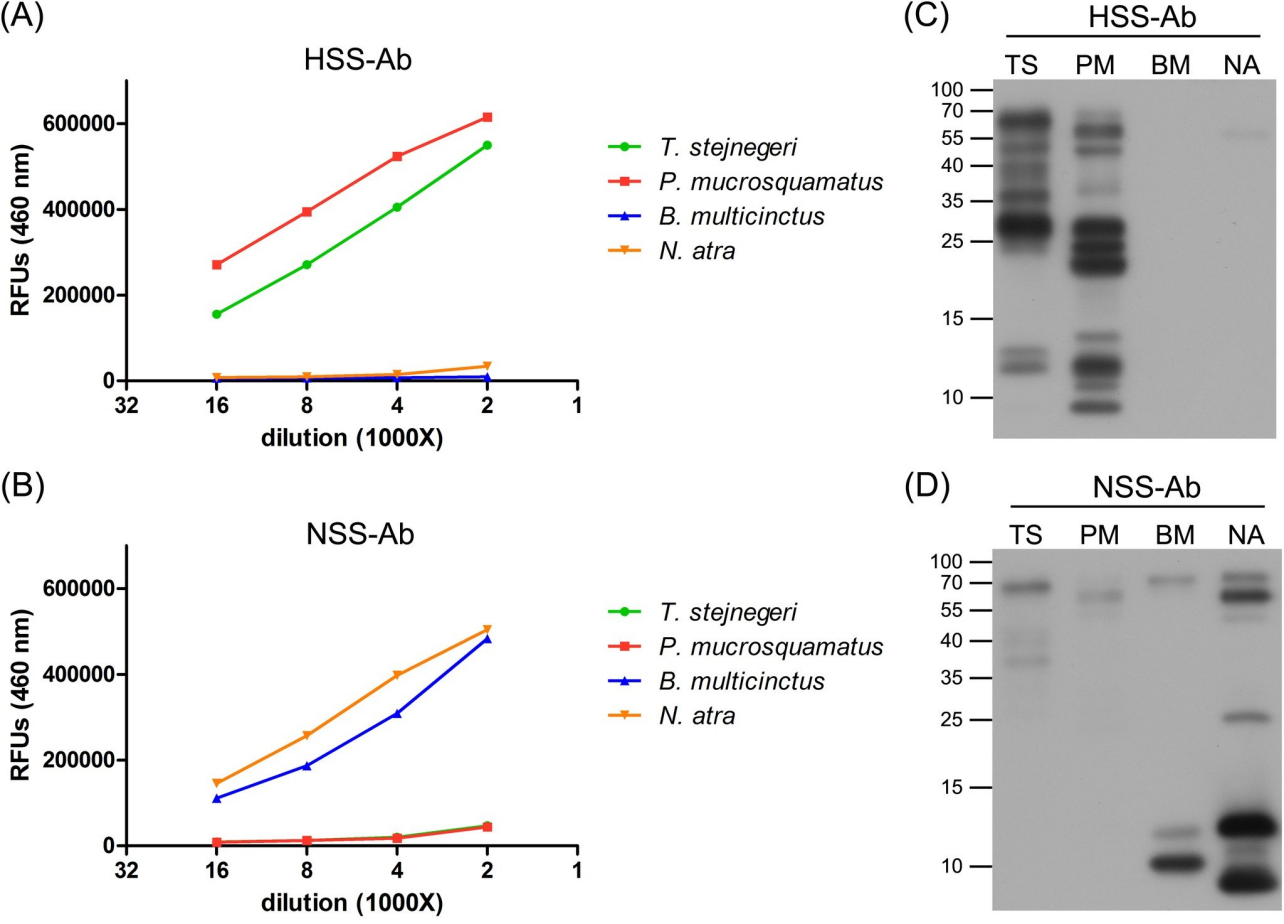
The specificity of HSS-Ab and NSS-Ab. (A, B) HSS-Ab (A) and NSS-Ab (B) were serially diluted from 1:2000 to 1:16000 and used as primary antibodies to detect four snake venoms. The assay was performed in duplicate, and results are presented as average values in relative fluorescence units (RFUs). (C, D) Venom proteins (5μg) from *T*. *stejnegeri* (TA), *P*. *mucrosquamatus* (PM), *B*. *multicinctus* (BM), and *N*. *atra* (NA) were resolved by SDS-PAGE on 15% gels, transferred to PVDF membranes, and probed with HSS-Ab (C) and NSS-Ab (D) (2 μg/ml) as primary antibodies.

In this study, we used an affinity purification procedure to eliminate cross-reactive antibodies from antivenoms. Heterologous venom-immobilized affinity columns were prepared and used to remove cross-reactive antibodies from antivenoms, after which the remaining antibodies were purified using a homologous venom-immobilized affinity column, yielding SSAbs. SDS-PAGE analysis of the affinity-purified HSS-Abs and NSS-Abs showed a typical pattern of IgG heavy and light chains (**S2 Fig**). Indirect ELISAs and Western blotting assays were performed to evaluate the specificity of affinity-purified SSAbs, HSS-Abs and NSS-Abs. The results of indirect ELISAs showed that both SSAbs possessed high specificity toward the homologous venoms, and showed significantly decreased cross-reactivity with heterologous venoms compared with the original antivenoms (**Fig 2A & 2B**). The immunoreactivity of HSS-Ab towards *P*. *mucrosquamatus* venom was stronger than that towards *T*. *stejnegeri* venom, whereas NSS-Ab preferentially reacted with venom proteins from *N*. *atra* compared with those from *B*. *multicinctus*. Consistent with the ELISA data, Western blot analyses also showed the high specificity of HSS-Ab and NSS-Ab towards their homologous venoms (**Fig 2C & 2D**), although NSS-Ab did weakly react with high-molecular-weight proteins (55–70 kDa) in the two hemorrhagic venoms (**Fig 2D**). Proteins in the high molecular weight region (25–70 kDa) of *T*. *stejnegeri* and *P*. *mucrosquamatus* venom represented the dominant targets of HSS-Ab (**Fig 2C**); in contrast, NSS-Ab mainly recognized lower molecular weight proteins (<15 kDa) in the two neurotoxic venoms (**Fig 2D**).

### Development of sandwich ELISA assays for detecting four snake venoms

**Fig 3:**
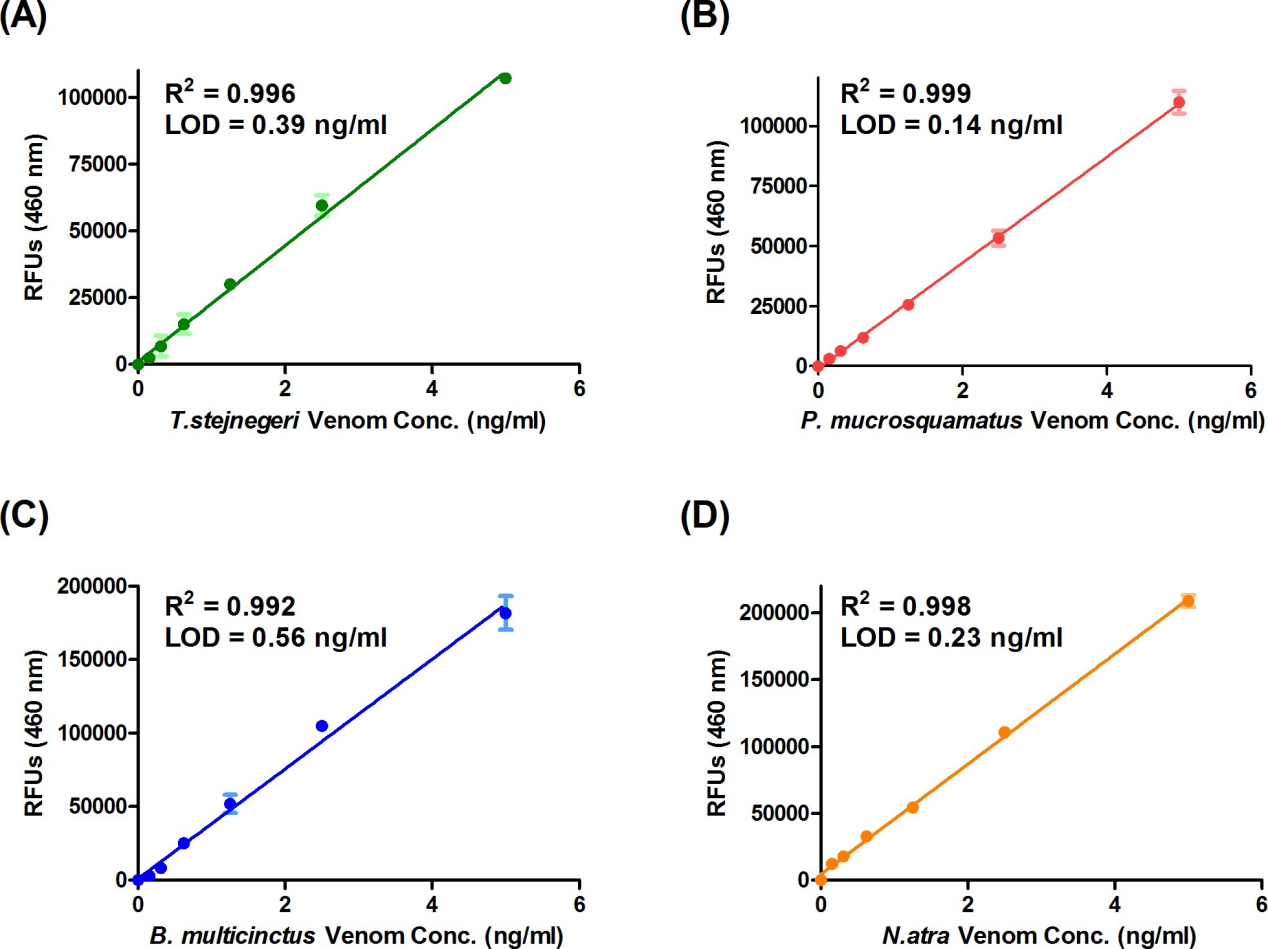
Calibration curves of sandwich ELISAs for detecting four snake venoms. Serially diluted venom proteins from (A) *T*. *stejnegeri*, (B) *P*. *mucrosquamatus*, (C) *B*.*multicinctus* and (D) *N*. *atra* were subjected to HSS-Ab–based (A and B) or NSS-Ab–based (C and D) sandwich ELISAs in triplicate. Shown here are the assay results used to generate the standard curves for measuring venom protein concentrations in snakebite patients.

To form sandwich complexes for ELISA measurements, we used HSS-Ab (or NSS-Ab) as the capture antibody and biotinylated HSS-Ab (or NSS-Ab) as the detection antibody. Antibody concentrations, buffers, and incubation times used for these sandwich ELISAs were optimized based on the ELISA development guide provided by the manufacturer (R&D Systems, Inc.). To determine the sensitivity of sandwich ELISA assays for snake venom detection, we serially diluted the four snake venoms in plasma and measured their reactivity by sandwich ELISA, generating standard curves for each venom (**Fig 3**). The limits of detection (LODs) of sandwich ELISAs for detecting *T*. *stejnegeri*, *P*. *mucrosquamatus*, *B*. *multicinctus* and *N*. *atra* venom were 0.39, 0.14, 0.56 and 0.23 ng/ml, respectively. In all cases, R^2^ values of standard curves were greater than 0.99. Taken together, these results suggest that our sandwich ELISA has the potential to identify snake species and quantify venom proteins in body fluids. For further application of this snakebite sandwich ELISA, the four venoms were used as the gold standards for venom quantification, and the LOD value determined as described above was set as the cutoff for detecting each venom.

**Fig 4:**
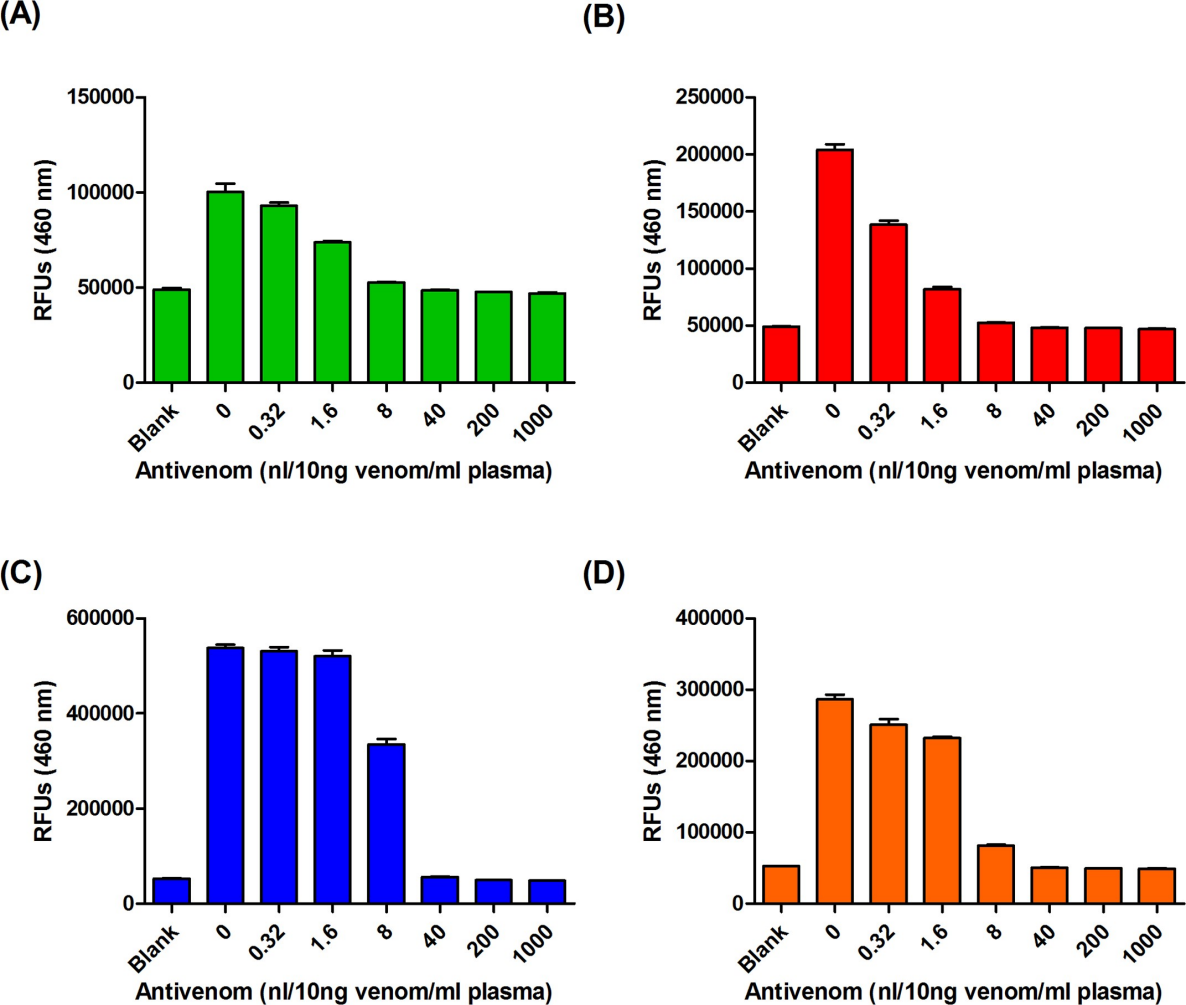
Pre-incubation of venom-containing human plasma with FHAV or FNAV diminishes HSS-Ab–and NSS-Ab–reactive ELISA signals. (A, B) Venom proteins of (A) *T*. *stejnegeri* and (B) *P*. *mucrosquamatus* were diluted in human plasma (10 ng venom protein per ml of plasma) and then mixed with serially diluted FHAV (0.32 to 1000 nl) at room temperature for 30 min. The mixtures were then subjected to HSS-Ab–based sandwich ELISA assay. (C, D) Venom proteins of (C) *B*. *multicinctus* and (D) *N*. *atra* were diluted in human plasma (10 ng venom protein per ml of plasma) and then mixed with serially diluted FHAV (0.32 to 1000 nl) at room temperature for 30 min. The mixtures were then subjected to NSS-Ab–based sandwich ELISA assay.

To determine whether snake venoms are still detectable after neutralization by antivenoms, we individually neutralized a fixed amount of venom with serially diluted antivenoms and then performed sandwich ELISAs. ELISA signals produced by 10 ng of *T*. *stejnegeri* (**Fig 4A**) and *P*. *mucrosquamatus* (**Fig 4B**) venom were completely eliminated by 8–40 nl of FHAV. Similarly, 40–200 and 8–40 nl of FNAV totally blocked ELISA signals derived from 10 ng of *B*. *multicinctus* (**Fig 4C**) and *N*. *atra* (**Fig 4D**) venom, respectively. These observations show that our sandwich ELISA assays only detects “free” venom proteins, and not antivenom-neutralized venoms. Importantly, they also suggest that our assays are suitable for evaluating the amount of free venom proteins remaining in a snakebite victim, making it possible to determine whether the dosage of antivenom delivered is sufficient to treat the patient.

### Venom detection by sandwich ELISA in an experimental envenomation animal model

**Fig 5:**
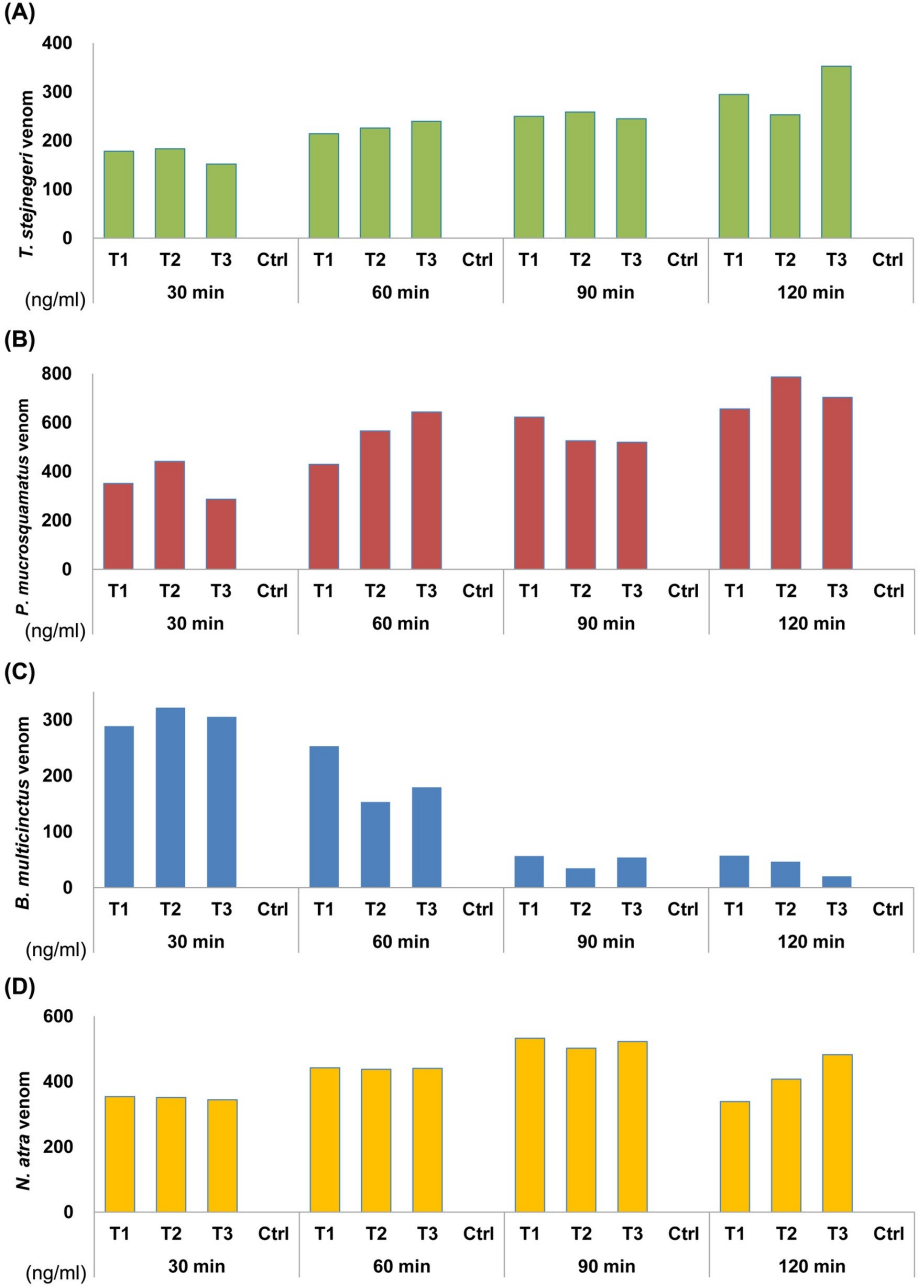
Detection of snake venom proteins in plasma samples from an animal model of snakebite. Each mouse was injected with the MLD of venom proteins from (A) *T*. *stejnegeri*, (B) *P*. *mucrosquamatus*, (C) *B*. *multicinctus* or (D) *N*. *atra*, and plasma samples were collected from venous blood at four time-points (30 min, 1 h, 1.5 h and 2 h) post injection. Venom concentrations were determined by analyzing the collected plasma samples using HSS-Ab–based (A and B) or NSS-Ab–based (C and D) sandwich ELISAs.

The MLD of each venom was determined using an experimental envenomation animal model. The MLD of *T*. *stejnegeri*, *P*. *mucrosquamatus*, *B*. *multicinctus* and *N*. *atra* were 1.5, 3, 0.3 and 0.65μg/g, respectively. All mice developed local symptoms within 10–20 min after injection of a lethal dose of venom. As soon as 30 min post injection, all four venoms could be detected by sandwich ELISA in plasma samples from mice injected with venom; as expected, none of the saline-injected control mice showed a positive reaction in these assays (**Fig 5**). The plasma concentrations of *T*. *stejnegeri* (**Fig 5A**), *P*. *mucrosquamatus* (**Fig 5B**) and *N*. *atra* (**Fig 5D**) venom proteins in these mice gradually increased during a 2-h period post injection. In contrast, the plasma concentrations of venom proteins in mice injected with *B*. *multicinctus* venom decreased dramatically during this period (**Fig 5C**). Collectively, these results demonstrate that the newly developed sandwich ELISA can successfully identify and quantify these four Taiwanese snake venoms *in vivo*.

### Assay performance of lateral flow strips

**Fig 6:**
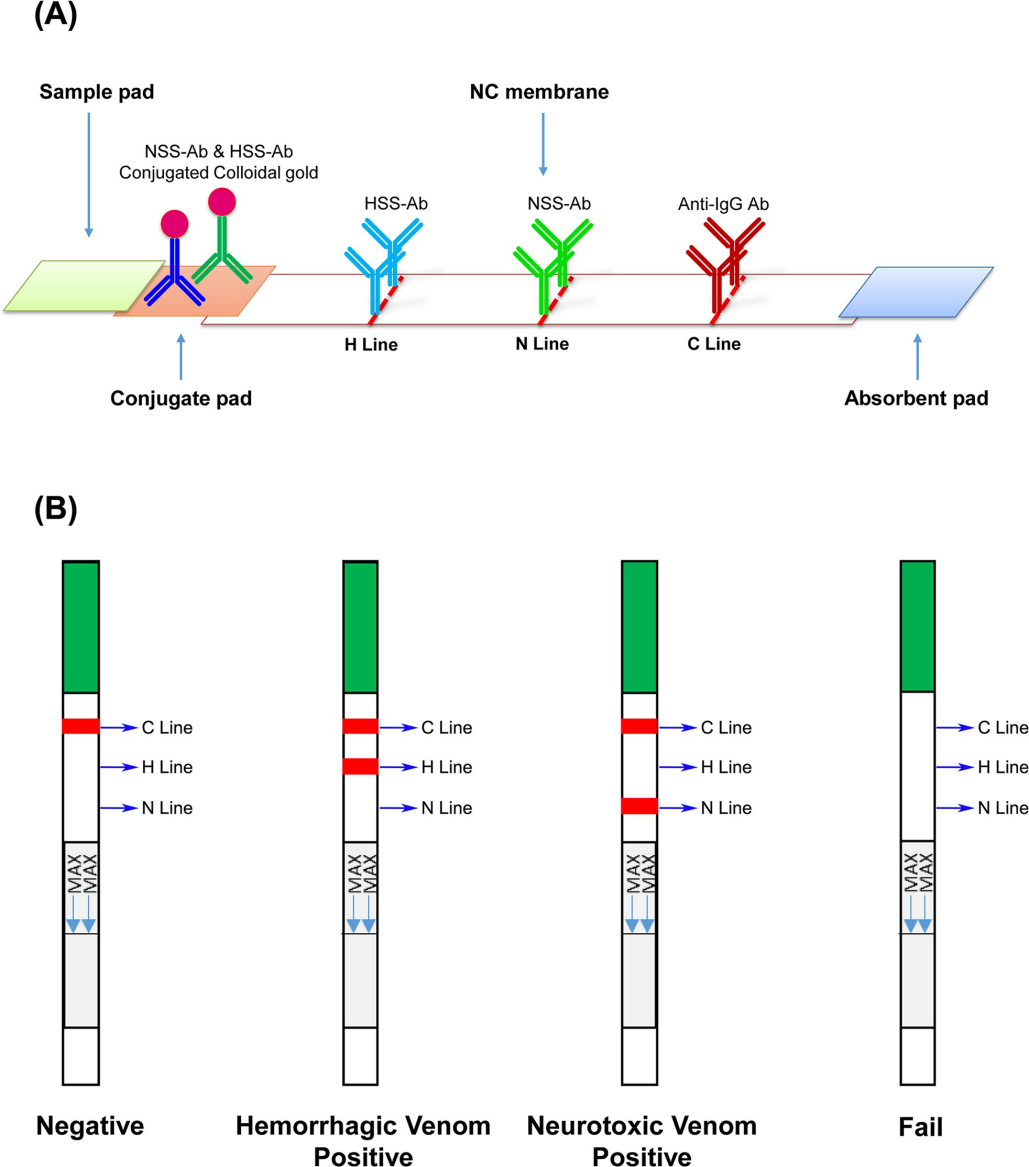
Schematic diagram of the lateral flow strips for detecting snake venom proteins. (A) The designof the lateral flow strip constructed on the basis of HSS-Ab and NSS-Ab and (B) a schematic depiction of predicted results are shown. C line, control line; H line, hemorrhagic test line; N line, neurotoxic test line.

**Fig 7:**
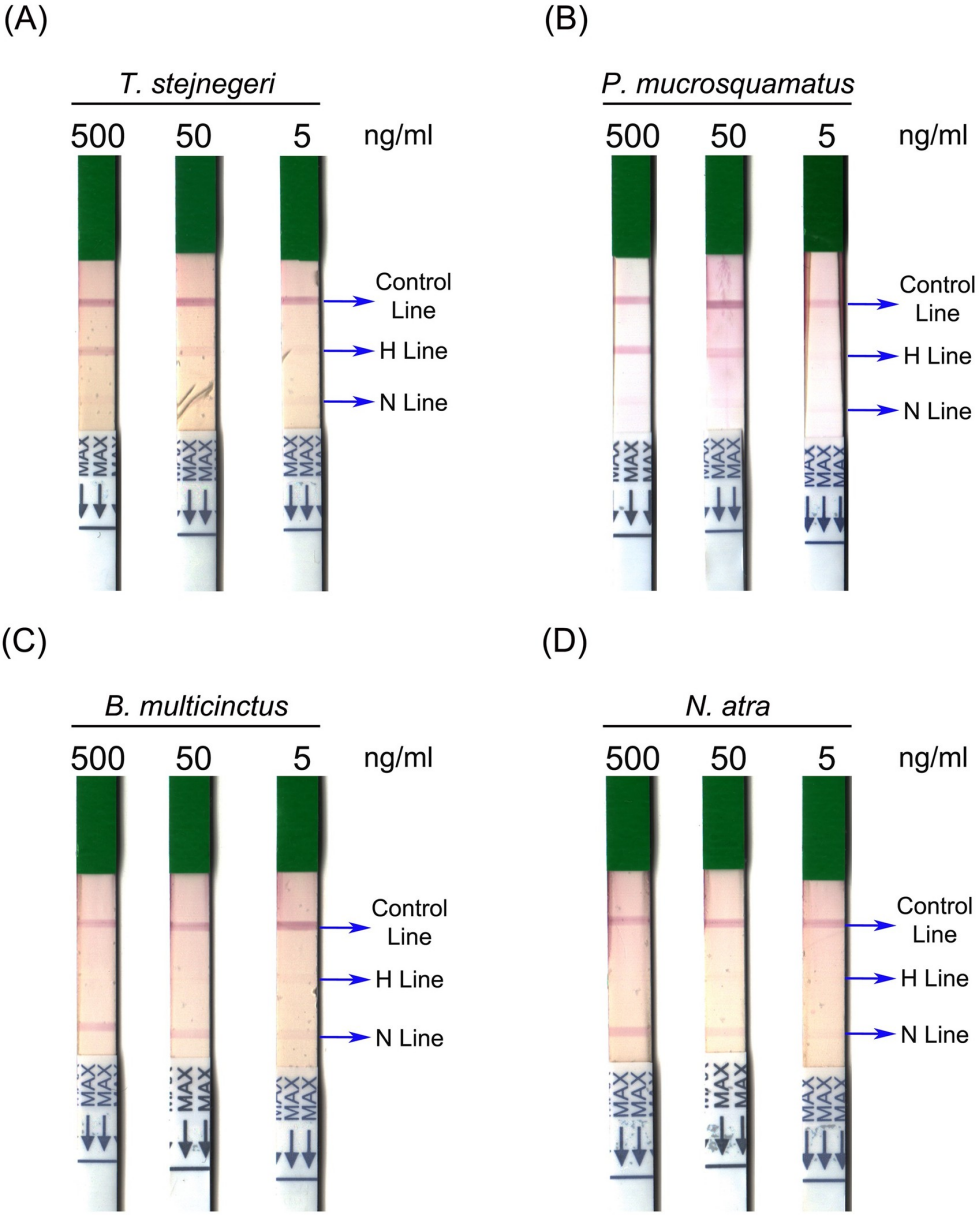
Performance of lateral flow strip assays in the detection of four snake venoms. Venom proteins of (A) *T*. *stejnegeri*, (B) *P*. *mucrosquamatus*, (C) *B*. *multicinctus* and (D) *N*. *atra* were serially diluted to 500, 50 and 5 ng/ml in human plasma, and then subjected to the lateral flow strip assay.

Although the newly developed sandwich ELISA assay exhibited high specificity and sensitivity, the assay time in its current format is too long for use in clinical practice. To reduce the operation time and simplify the platform for snakebite diagnosis, we sought to develop another assay using a lateral flow strip format with two test lines (**Fig 6A**). To assess the specificity and sensitivity of this lateral flow strip, we tested it on the four venoms serially diluted (from 500 ng/ml to 5 ng/ml) in human plasma. The assay was evaluated based on the appearance of a control line, a hemorrhagic test line (H line), or a neurotoxic test line (N line) (**Fig 6B**). All strips showed a visible control line, confirming that all test samples were successfully flowed onto the strips (**Fig 7**). An H line was only observed in those strips used to test *T*. *stejnegeri* and *P*. *mucrosquamatus* venom (**Fig 7A & 7B**), and the N line appeared only in assays of *N*. *atra* and *B*. *multicinctus* venom proteins (**Fig 7C & 7D**). These results indicate that this newly developed strip assay does not exhibit sufficient cross-reactivity to cause ambiguous results. In assays of hemorrhagic venom, the H line was still detectable after reducing the concentration of *T*. *stejnegeri* and *P*. *mucrosquamatus* venom proteins to 50 ng/ml (**Fig 7A & 7B**). For neurotoxic venom detection, the N line was still visible when both venom protein levels were reduced to 5 ng/ml (**Fig 7C & 7D**).

### Venom detection in clinical samples by sandwich ELISA and lateral flow strip assays

Thirty-two victims of snakebite sent to Emergency Departments of the four participating hospitals from May 2017 to February 2018 were enrolled in this study. Among them, eleven patients were excluded because they had been treated with the appropriate antivenom before arrival in the Emergency Department (n = 9) or displayed no symptoms (n = 2). The serum samples obtained from the remaining 21 cases were analyzed by sandwich ELISA and lateral flow strip assay (**Table 1**). The lateral flow strip assay showed 100% (5/5) specificity and 100% specificity (5/5) for the detection of neurotoxic envenomation samples. However, the sensitivity for detecting hemorrhagic envenomation samples was only 36.4% (4/11). We used the kappa statistic to assess the strength of agreement between the two assays, and this analysis indicated good to fair agreement (κ = 0.53) between snakebite sandwich-ELISA and lateral flow strip assay (**Table 1**).

**Table 1:** Correlation between sandwich ELISAs and lateral flow strips for snakebite diagnosis.

Lateral flow strip	ELISA				Total
	FH (+)	FN (+)	Negative	Kappa	
FH (+)	4	0	0	0.53	4
FN (+)	0	5	0		5
Negative	7	0	5		12
Total	11	5	5		21

The clinical information of these 21 patients were summarized in **Table 2**. Most of the culprit snakes were initially identified by patients’ description or recognition of snake photograph (17/21), and 2 of them were definitely confirmed according to the killed snakes brought to the hospital. The aggressor snakes of case 18–21 cannot be identified at scenes of ED. In the laboratory identification, both ELISA and lateral flow strip assay were shown hemorrhagic venom positive results for case 18 and 19, and venom negative result for case 20 and 21. All patients were presented with local swelling except case 11 who was initially identified as *B*. *multicinctus* envenomation, and no neurologic symptoms appeared in all. Case 16, 18 and 19, who were performed surgery, have higher level of venom concentration than other victims. Seven cases with hemorrhagic venom-positive ELISA results appeared with negative lateral flow strip results. The venom concentration of them was ranged from 2.2 to 10.6 ng/ml, which are lower than other cases detected by lateral flow strip assay. Among them, five cases were shown mild clinical severity, and 2 cases shown moderate severity. Case 11, 15, 17, 20 and 21 have ELISA undetectable venom level. All of them have mild clinical severity that the local swelling restricted in fang mark area, or even did not have local swelling. The sample time after bite for the majority of the victims (15/21) was ≦3.5 h. Overall, there was no significant correlation between the blood venom concentration and sampling time after snakebite or the bitten area according to this small-scale clinical study.

**Table 2:** The clinical information of 21 snakebite cases enrolled in this study.

Initial identification^a^	Case No.	Sex	Sampling time after bite (h)	Bitten area	Local swelling	Surgery	Clinical severity ^b^	Antivenom dosage	ELISA	Lateral flow strip	
									Venom Conc. (ng/ml)	FH	FN
*T*. *stejnegeri*	1	M	1	Foot	Foot	-	Mild	1 FHAV	3.2 (TS)	**-**	**-**
	2	M	1.5	Hand	Forearm	-	Moderate	1 FHAV	2.2 (TS)	**-**	**-**
	3	F	0.5	Finger	Finger	-	Mild	2 FHAV	10.6 (TS)	**-**	**-**
	4	M	1	Finger	Forearm	-	Moderate	5 FHAV	19.7 (TS)	**+**	**-**
*P*. *mucrosquamatus*	5	F	6	Wrist	Elbow	-	Moderate	1 FHAV	3.9 (PM)	**-**	**-**
	6	M	1	Toe	Calf	-	Moderate	2 FHAV	141 (PM)	**+**	**-**
	7	F	0.5	Ankle	Ankle	-	Mild	2 FHAV	9.1 (PM)	**-**	**-**
	8	F	1.5	Finger	Hand	-	Mild	1 FNAV	14.3 (NA)	**-**	**+**
	9*	M	1	Ankle	Ankle	-	Mild	1 FHAV	2.6 (PM)	**-**	**-**
	10	F	0.5	Foot	Ankle	-	Mild	1 FHAV	6 (PM)	**-**	**-**
*B*. *multicinctus*	11	M	14	Finger	-	-	Mild	1 FNAV	ND ^c^	**-**	**-**
*N*. *atra*	12	M	3.5	Foot	Ankle	-	Mild	-	21.2 (NA)	**-**	**+**
	13*	M	1	Foot	Foot	-	Mild	2 FNAV	93.6 (NA)	**-**	**+**
	14	M	1	Finger	Wrist	-	Moderate	5FNAV	147.3 (NA)	**-**	**+**
	15	M	10.5	Toe	Toe	-	Mild	-	ND	**-**	**-**
	16	M	1.5	Toe	Ankle	Debridement	Severe	4 FNAV	297.8 (NA)	**-**	**+**
	17	M	1.5	Finger	Finger	-	Mild	4 FNAV	ND	**-**	**-**
Unidentified	18	M	12.5	Finger	Shoulder	Debridement/Fasciotomy	Severe	4 FHAV	210.6 (TS)/77.6 (PM)	**+**	**-**
	19	M	Unknown	Forearm	Upper arm	Debridement/Fasciotomy	Severe	4 FHAV	90.3 (TS)/59.4 (PM)	**+**	**-**
	20	M	2.5	Ankle	Ankle	-	Mild	1 FHAV	ND	**-**	**-**
	21	M	34	Thumb	Forearm	-	Mild	2 FHAV	ND	**-**	**-**

^a^ Initial identification: it is shown the envenoming snake species which was initially identified by patients’ description or recognition of snake photograph.

^b^ Clinical severity: the definition of each level of severity is shown in Supplemental Table 1.

^c^ Non-detected: The level of venom concentration is lower than the LOD of ELISA, or even no venom is existed in the clinical sample.

*: The envenoming species was confirmed from the culprit snake brought to the ER.

## Discussion

The presence of common antigens in heterologous venoms has been demonstrated to be a major source of bias for the development of snakebite detection assays [26, 42]. The appearance of widespread cross-reactivity between heterologous snake venoms and polyvalent or monovalent antivenoms considerably hampers the specificity of such assays [11, 12, 28, 43]. Consistent with these previous observations, the current study also found that FHAV and FNAV cross-reacted towards heterologous venoms, as evidenced by the detection of 3–5 protein bands in Western blot analyses (**Fig 2A & 2B**). However, snake venoms are known to comprise multiple (10–100) proteins, many of which have the same or similar epitope(s), but with different molecular weights. At present, it is difficult to predict the venom components that contribute to this cross-reactivity. Immunoaffinity purification appears capable of removing antibodies in antiserum that recognize common epitopes of venom components. Even though the identity of the species-specific antigens and common epitopes that contribute to the cross-reactivity remain largely unknown, we were still able to successfully obtain venom protein antibodies with high specificity (i.e., low cross-reactivity among different snake species). In addition, detection of snake envenomation by monoclonal antibodies generated using a single species-specific venom protein can considerably improve assay specificity [44–47]. However, the sensitivity of these antibodies may not be high enough, because venoms contain numerous protein components and a mAb can only react with a single epitope on its target protein. Moreover, the targeted venom component may become degraded through metabolic processes in biological systems. Thus, the application of monoclonal antibodies to the development of snakebite kits remains a considerable challenge. The promising data shown in the present study suggest that purification of SSAbs from antivenoms could be a feasible and cost-effective strategy for generating effective probes for snake venom detection and species discrimination.

Sandwich ELISAs, which have been widely used in snake venom detection and snakebite diagnosis [10, 11, 44, 48], are capable of measuring venom proteins at the level of a few nanograms per milliliter. In conjunction with the biotin-streptavidin amplification system, the detection limit can be further improved, reducing the lower limit to less than 1 ng/ml [10]. Generally, two different antibodies are used for sandwich ELISA assay development. Because we used the same SSAb as both capture and detection antibody in our sandwich ELISA, the capture SSAbs in the solid phase only occupied one binding site on their cognate antigen molecules. Thus, the detection SSAb was still capable of recognizing the remaining epitopes on the captured antigens. With this approach, how to pair two suitable antibodies to form the sandwich complex for detection is not a concern, making it easy to adapt for snake venom detection. Although the sandwich ELISA assay is time consuming, and thus is likely not the most appropriate assay for use in emergency rooms, it is still a good tool for snakebite epidemiology and prognosis studies.

The usefulness of our sandwich ELISA assay was demonstrated by detecting venoms in blood samples from an experimentally envenomed mouse model (**Fig 5**). These experiments showed that this assay is capable of identifying the envenoming species and quantifying venom concentrations in blood. Application of this ELISA to the snakebite animal model revealed that concentrations of *T*. *stejnegeri*, *P*. *mucrosquamatus* and *N*. *atra*venom proteins gradually increased in mouse plasma during a 2-h period post-injection; in contrast, the concentration of *B*.*multicinctus* venom proteins dramatically decreased over this same time period (**Fig 5**). A previous study reported that more than half (nearly 60–80%) of *B*.*multicinctus* venom components are neurotoxins, including β-bungarotoxin,α-bungarotoxin and γ-bungarotoxin [49]. These bungarotoxins bind to specific receptor(s) on presynaptic and postsynaptic membranes, leading to paralysis and neurotoxicity [50–52]. Our findings suggest that, when injected into the victim, these bungarotoxins rapidly interact with specific receptors, and thus are immobilized in the neuromuscular junctions; this, in turn, causes a significant decrease in their bioavailability, accounting for the rapid decrease in their concentration in blood plasma.

The lateral flow strip assay is a sandwich-based immunostrip used to rapidly (5–20 min) examine whether target molecules are present in a sample [53]. This type of assay is appropriate for use in snakebite detection and diagnosis, and can offer guidance to physicians in administering antivenom [22, 23]. Furthermore, the visual diagnosis format of this assay is simple, making it desirable for use in developing countries, where snakebites are most prevalent. However, some factors and sampling conditions may profoundly affect strip assay results. For example, a high concentration of serum proteins and high viscosity of the test sample could interfere with the formation of the red line in test and control zones, and samples containing high concentrations of salt, such as urine, often cause false-positive results. Thus, in some situations, sample pretreatment is required. The lateral flow strip assay developed here has two test lines for discriminating hemorrhagic and neurotoxic snake envenomation in Taiwan. This strip assay successfully detected and identified snake venom in serum samples from snakebite patients.

**Fig 8:**
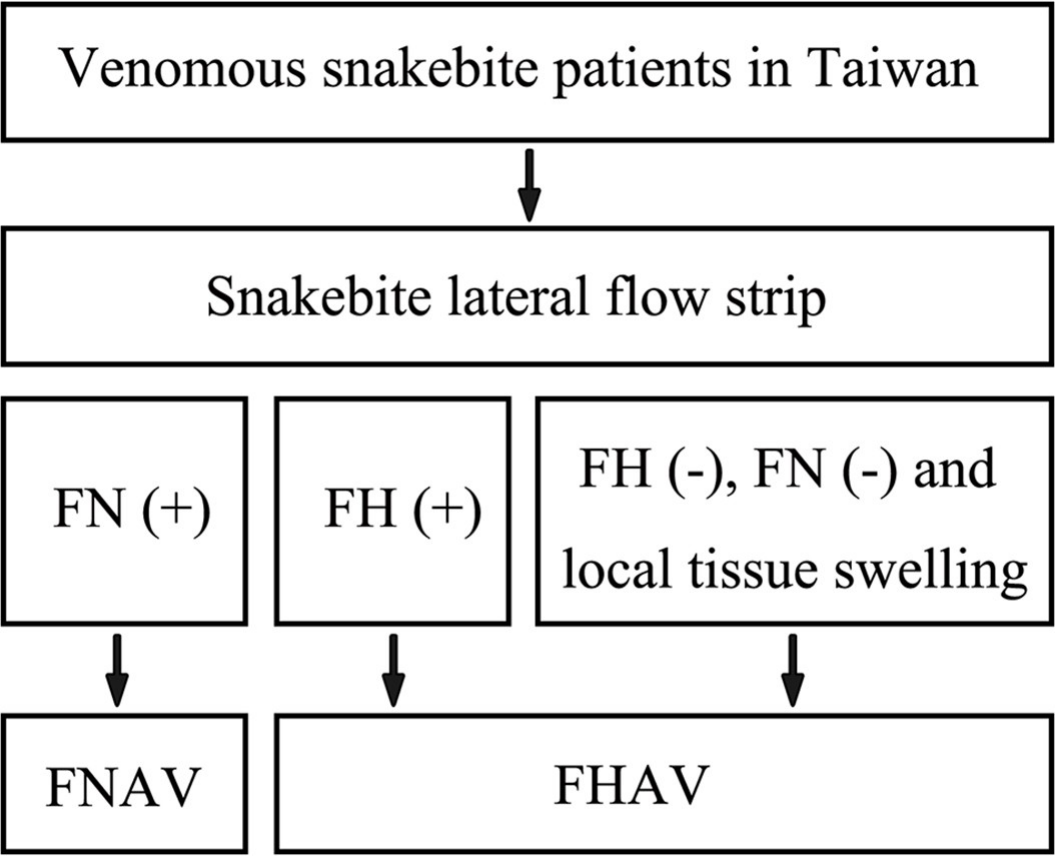
A proposed diagnosis flowchart for venomous snakebite patients in Taiwan by incorporating the newly developed lateral flow strip assay.

Our small-scale clinical study demonstrated that the lateral flow strip assay is useful for assessing neurotoxic envenomation, exhibiting a sensitivity/specificity of 100%. It is suggested that the newly developed strip assay holds promise for the diagnosis of neurotoxic snakebite. However, the sensitivity of this assay for hemorrhagic envenomation was nearly 40%. ELISA results of these 11 hemorrhagic envenomation samples showed that the *T*. *stejnegeri* or *P*. *mucrosquamatus* venom protein concentrations in 7 lateral flow strip-negative samples were less than 10 ng/ml (**Table 2**), suggesting that this assay is not sensitive enough to detect snakebite cases with low blood concentration of hemorrhagic venom in clinical practice. Although, at this point, we cannot definitively establish the appropriateness of our lateral flow strip assay for precise diagnosis of all clinical snakebites, the combination of clinical symptoms and the results of lateral flow strip could improve the clinical utility of our lateral flow strip, especially in the weak aspect of diagnosis of hemorrhagic snake envenomation. A diagnosis flowchart which composed of clinical symptoms and the result of lateral flow strip was therefore proposed (**Fig 8**). Because of the relative high sensitivity and specificity of our lateral flow strip in diagnosis of neurotoxic snake envenoming, cases with negative lateral flow strip results have a great possibility of hemorrhagic snake envenoming when they have developed local tissue swelling. This diagnosis flowchart may further enhance the ability of our lateral flow strip to guide the usage of antivenom. Because only 21 snakebite cases were included, further study using a larger sample set is needed to verify the sensitivity, specificity, stability, and feasibility of this strip assay.

Seven of the 21 clinical samples examined in this study showed positive ELISA result but negative on the lateral flow strip test. All of them were identified as hemorrhagic snake envenomation with low venom concentration level accompanying with mild or moderate clinical severity. Even though these patients have been transferred to hospital and sampled nearly within 1–2 hrs, their blood venom concentrations were still lower than the others. It is highly possible that the amount of venom injected into these victims was originally low, which is hard to detect by lateral flow strip assay after dilution in the systemic circulation, and only induced mild clinical symptoms. Despite initial identification of envenoming species is almost the same as the test results in our small-scale study (**Table 2**), sometimes, envenoming species identified by patients or their family may mislead the physicians. Take case 8 as an example, this patient was initially identified as *P*. *mucrosquamatus* envenomation according to family members’ recognition of the snake pictures, however, both ELISA and lateral flow strip assay showed positive result of neurotoxic snake envenomation, indicating the culprit snake is *N*. *atra*. Furthermore, few cases with negative result of both assays may be bitten by non-venomous snakes. There are more than 50 snake species in Taiwan. It is hard for citizens to correctly recognize and distinguish all of them. Bringing the envenoming snake to the hospital, like cases 9 and 13, is the most reliable way for species identification.

In the present study, all five cases (case 11, 15, 17, 20 and 21) with negative quantification of venom displayed the mild clinical severity. These patients may be bitten by non-venomous snakes, or the dry bite. As mentioned above, snakebite victims have the chance to misidentify the envenoming species, and slight swelling usually occurred around the fang mark even if they were bitten by non-venomous snakes. It is one of the reasons leading to the negative results in both assays. In addition, although we did not observe a close relationship between the transcurrent time from the bites to the ER consult and the results of the diagnostic test, 3 of the 5 cases with negative ELISA result had longer transcurrent time. Case 11, 15 and 21 had their transcurrent time for 14, 10.5 and 34 hrs, respectively. The metabolism time more than 10 hours may allow the venom to be eliminated from patients’ body and resulted in negative test result. The delay in seeking medical help may be another reason leading to the negative test results. On the other hand, case 18 had 12.5 hours of transcurrent time, but displayed severe clinical symptoms and positive test results. It is reasonable to assume that the type and amount of venom injected into patients is the main factor to determine the outcome of the test results, and the effect of transcurrent time could be minor.

The current study used serum samples from snakebite patients to evaluate the performance of the snakebite lateral flow strip assay. Other types of specimen, such as urine, wound exudate and blister fluid, have been reported as alternatives for venom detection [7, 12]. The highest amounts of venom proteins (>100 ng/ml) are found in wound exudates and blister fluid; thus, venom proteins are more easily detected and measured in these types of specimens [12]. However, cases with blister fluid are very rare; in the current study, only one patient formed blister fluids after envenomation. Wound exudates are easier to obtain than blister fluids, but obtaining untreated wound exudates for pre-clinical trials is another challenge. Because people have been taught to perform first aid when bitten by snakes, snake venom remaining in the wound will typically have been washed out or swabbed out. Furthermore, fang marks have usually clotted by the time victims arrive at the Emergency Department. Thus, although wound exudate maybe the best sample type for venom detection, how to collect good quality samples for survey remains a daunting challenge.

Countries in tropical and subtropical regions have various indigenous venomous snake species. Two or more antivenoms are currently available for clinical treatment of snake envenomation. Directly using these antivenoms as a resource for the development of snakebite diagnostic assays could be a cost-effective approach for snakebite management. The use of an affinity purification strategy makes it possible to obtain SSAbs from antivenoms, thereby eliminating cross-reactive antibodies and preventing false-positive results in assays of snake venoms. This approach obviates the need to produce additional polyclonal or monoclonal antibodies, and alleviates concerns regarding whether the antigens targeted by the polyclonal or monoclonal antibodies produced are species specific. SSAbs purified from antivenoms are suitable for use in developing sandwich ELISAs and lateral flow assays for rapid detection of snake venoms. The ability of these purified SSAbs to detect venom in the blood of animal models as well as in blood samples taken from snakebite patients validates the usefulness of this strategy.

In conclusion, our data indicate the feasibility of a cost-effective approach (i.e. preparation of SSAbs from specific antivenoms available in Taiwan) to develop the snakebite diagnostic assay for discriminating hemorrhagic and neurotoxic snake envenomation in Taiwan. When combining the clinical observation of patient’s symptom, this assay would aid in the clinical decision of the appropriate antivenom to be used where the signs and symptoms of the envenoming did not allow a precise diagnosis by the clinician responsible to treat the envenomed patient. Although our present results are promising, further studies including improvement of detection sensitivity/specificity of the assays and application of the optimized assays to a larger sample set are needed to validate the clinical utility of the assays for snakebite management.

## Supporting information

S1 Fig Protein profiles of the four snake venoms. Venom proteins (5 μg) from each of the four snakes were resolved by SDS-PAGE on 15% gels and visualized by Coomassie blue staining.
(TIF)

S2 Fig SDS-PAGE analysis of affinity-purified HSS- and NSS-Abs. Affinity-purified HSS-Abs and NSS-Abs (1 ml for each) were resolved by SDS-PAGE on 15% gels and visualized by Coomassie blue staining.
(TIF)

S1 Table The definition of Clinical severity of Snakebite. (XLSX)
