# Supplementary figures and images for: Development of sandwich ELISA and lateral flow strip assays for diagnosing clinically significant snakebite in Taiwan
Source: PLoS Negl Trop Dis. 2018 Dec 3;12(12):e0007014. doi: 10.1371/journal.pntd.0007014 (PMC6292642; doi:10.1371/journal.pntd.0007014)

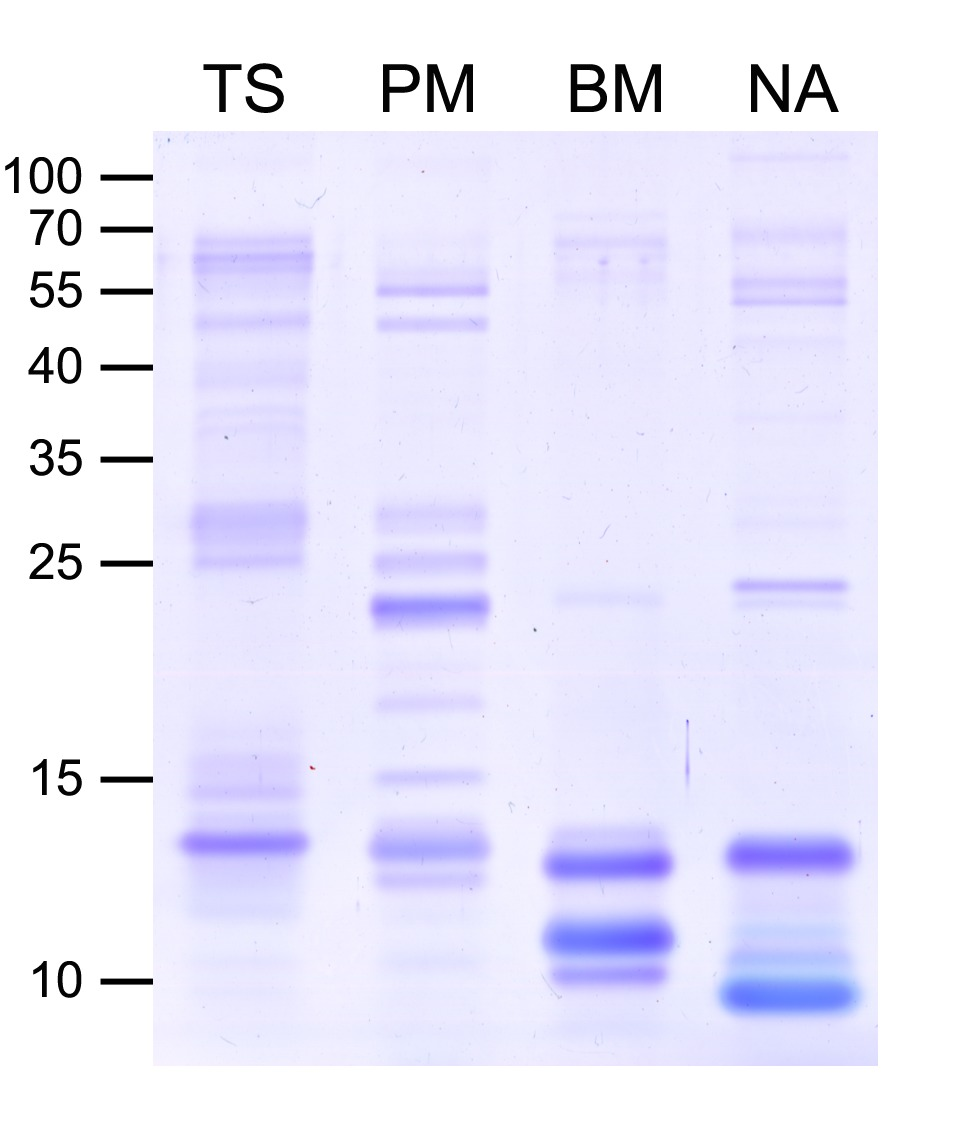

Supplement: S1 Fig — Venom proteins (5 μg) from each of the four snakes were resolved by SDS-PAGE on 15% gels and visualized by Coomassie blue staining. (TIF) [file pntd.0007014.s001.tif]

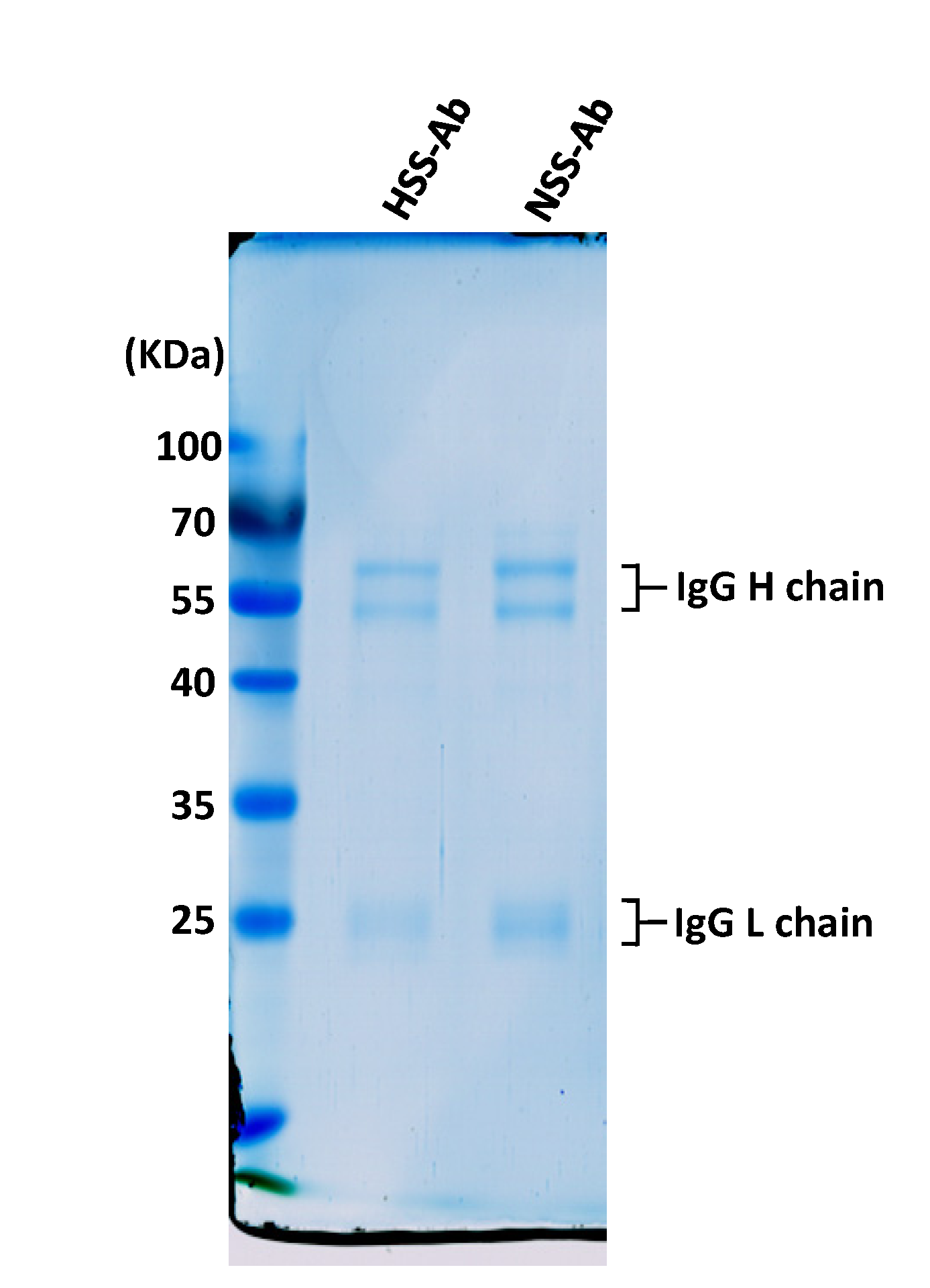

Supplement: S2 Fig — Affinity-purified HSS-Abs and NSS-Abs (1 ml for each) were resolved by SDS-PAGE on 15% gels and visualized by Coomassie blue staining. (TIF) [file pntd.0007014.s002.tif]
